# Analysis of Structure and Function of Ladybird Leg and Subsequent Design and Fabrication of a Simplified Leg Structure for Robotic Applications

**DOI:** 10.3390/biomimetics9030184

**Published:** 2024-03-18

**Authors:** Christopher Mercer, Naoe Hosoda

**Affiliations:** Smart Interface Group, Research Center for Structural Materials, National Institute for Materials Science, Tsukuba 305-0044, Japan

**Keywords:** ladybird leg, bio-inspired, 3-D printing, tendon configuration

## Abstract

Many insects are able to walk vertically or upside down on both hard and soft surfaces. In beetles such as the ladybird (*Coccinella septempunctata*), intermolecular forces between tarsal setae on the footpads of the insects make this movement possible. In prior work, adhesion structures made from polydimethylsiloxane (PDMS) that mimic the action of the tarsal setae have been developed. It is proposed that these adhesion structures could be attached to a simplified version of the leg of a ladybird and used in practical applications. For example, the leg structures could potentially be employed in small surveillance drones to enable attachment to surfaces during flights, in order to preserve battery power. Alternatively, the structures could be used in small robotic devices to enable walking on steeply inclined surfaces. In this program of work, the morphology and movement of the leg of a ladybird were closely studied using a 3D X-ray microscope and a high-speed microscope. The positions of the tendons that facilitated movement were identified. From this knowledge, a simplified leg structure using pin-joints was designed and then fabricated using 3-D printing. The PDMS adhesion structures were then attached to the leg structure. The tendons in the actual insect leg were replicated using thread. Typical detachment forces of about 4 N indicated that the simplified leg structure was, in principle, more than capable of supporting the weight of a small device and then detach successfully. Attachment/detachment movement operations were performed using a linear actuator and controlled remotely. Therefore, proof of concept has been demonstrated for the use of such a simplified ladybird leg structure for the attachment/detachment of small robotic devices to horizontal, inclined, or vertical surfaces.

## 1. Introduction

**Figure 1:**
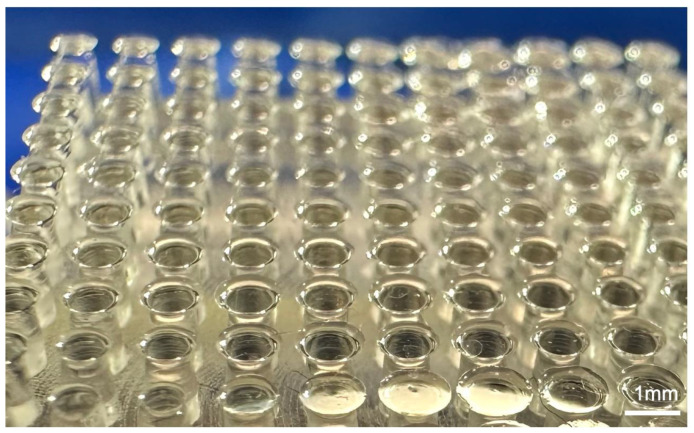
Polydimethylsiloxane (PDMS) adhesion structure that mimics tarsal setae on *Coccinella septempunctata*.

Insects such as the ladybird beetle (*Coccinella septempunctata*) exhibit a remarkable ability to walk upside down or on vertical surfaces [1,2,3]. Intermolecular forces between hair-like setae on the underside of the insect’s tarsus and the substrate generate sufficiently strong attachment to allow for this movement [2,4]. Prior research on the attachment mechanism has led to the development of adhesion structures that are similar in nature to the hairy structures found on the insect footpad. These artificial structures are fabricated from polydimethylsiloxane (PDMS) [5], carbon nano tube (CNT) [6], polyurethane [7], polyvinyl siloxane [8], polymethyl methacrylate resin (PMMA) [9], etc. and have been discussed in detail in References [10,11]. However, briefly summarized, the combination of the morphology of the structure and the material allows for attachment to smooth and flat substrates using molecular forces in a similar manner to the function of the setae on the insect tarsus. An image of an adhesion structure is shown in Figure 1. In addition, a complex articulation system of the legs of such insects facilitates detachment of the tarsus from the substrate as the insect walks [12]. It is envisioned that the mechanism of adhesion together with the configuration of a ladybird leg and the associated movement mechanisms could be employed in real-world applications. One possible area where the structures could potentially be of use is that of small robotic devices. Essentially, the PDMS, etc. adhesion structures would enable such a device to attach to steeply inclined or even vertical surfaces. Then, a replication of the insect leg could be used to facilitate detachment from the surface.

Studies on the tendons/muscles that move the tarsomeres and claws have been carried out on flies [12,13], stick insects [14,15], and hornets [16], as well as *Pachnoda marginata peregrina* [17]. In particular, the tendons that control claw movement have been studied in detail. Attachment organs, such as arolium, pulvilli, and euplantulae, lack specialized muscles controlling their movements. They are controlled together with other structures of the tarsus and pretarsus by a set of muscles located in the tibia and femur [16]. Insects with multiple tarsi, such as ladybirds and leaf beetles, are likely to have more complex movements of tarsi during walking, and it is unlikely that a single tendon can control complex movements as in other insects. Therefore, this study investigates the movement of tarsi and tendon structure during walking in ladybird beetles with multiple tarsi.

The study of biological leg and foot structures has great significance in the field of biomimetics, since such structures can be mimicked in a wide range of robotic applications. The structure and movement of both human and animal legs have been investigated in previous work [18,19,20,21,22,23,24,25,26,27,28,29,30]. Supernumerary robotic legs for human support [18] and locomotion [19] have been successfully demonstrated. Rezazadeh et al. [20] propose a constructive framework for both bio-inspired and mechanically optimized robotic legs. Xu et al. [21] achieved omni-directional locomotion of a robotic leg. In a similar study, Park et al. [22] constructed a robotic leg based on the domestic cat. Using the design, a biped robot could run at speeds of up to 0.75 mm/s. In addition, a significant amount of research has been performed on the structure and morphology of the feet of insects and geckos [23,24,25,26,27,28,29], in order to explain and replicate the remarkable adhesion ability that such animals exhibit. Lastly, some previous work has been performed on replicating the structure and movement of the legs of beetles specifically. Tran-Ngoc et al. [30] conducted a detailed investigation of the function and movement of the leg of the adult flower beetle, (*M. torquata*), and used this knowledge to fabricate a robotic leg based closely on the studied insect. The robotic leg was able to attach and then retract smoothly from a mesh substrate. However, in this study, a single tendon was used in the robotic leg. It is envisioned that the use of multiple tendons in robotic insect legs may provide additional functionality and could facilitate an even greater range of possible movements and substrate attachment/detachment operations.

The purpose of this work, therefore, was to conduct a detailed analysis of the morphology and articulation of a ladybird leg via 3D X-ray microscope and high-speed microscope evaluation techniques. Particular attention was given to the study of the location and configuration of the tendons that enable movement of the individual elements that comprise the tarsus of the ladybird beetle. In addition, observation of the movement of live insects provided insight into the mechanisms of detachment as the insect walked along the substrate. The acquisition of this knowledge was then used to design and fabricate (via 3-D printing) a simplified leg structure based on the real insect leg as discussed in the preceding paragraph. Artificial multiple tendons were used to move the leg elements. Automated movement can be achieved via battery-powered and remote-controlled linear actuation. From this work, the attachment/detachment of mechanical devices to/from horizontal, inclined, and vertical surfaces and even cylindrical surfaces can be realized.

## 2. Materials and Methods

### 2.1. Procurement of Insects

Ten adult ladybird beetles were collected from various plants (*Vicia angustifolia, Paeonia suffruticosa*, etc.) in the field in Tsukuba city, Japan (36°04′12″ N, 140°08′04″ E). The beetles were kept in small ventilated cages (70 × 70 × 100 mm^3^) at a temperature of 23–25 °C, and were fed with 5% sugar solution, which was provided soaked into cotton-wool balls.

### 2.2. Preparation of 3D X-ray Microscope Observation

Staining and dehydration treatments were carried out to observe the internal structure of ladybird legs. Legs were placed in a Farmer’s fixative (100% ethanol:glacial acetic acid = 3:1) for 2 h, followed by 15 min each in different ethanol concentrations (75%, 80%, 90%, 100%). The 100% ethanol solution was then added with 1% iodine and left for 2 days. It was dried on filter paper for 1 min. It was then placed in hexamethyldisilazane (HDMS) for 1 h and left in a fresh solution (HDMS) for 1 day.

The internal structure of ladybird legs was observed using a 3D X-ray microscope (Xradia 520 Versa, ZEISS, Jena, Germany).

### 2.3. Observation of Movement of the Tarsal Segments of the Ladybird Beetle

The beetle was anesthetized with carbon dioxide, and a thread was glued on the elytra with a droplet of melted wax. The beetle was guided to the arena of a horizontal glass plate and allowed to walk freely on it. During walking, the movement of tarsal segments was recorded from the side using a high-speed microscope (VW-9000, Keyence corporation, Osaka, Japan) (250 fps, ×50 or ×100).

The order of each tarsal segment contacting or detaching the glass plate during walking was observed and classified into seven sequential patterns for each behavioral event.

## 3. Relationship between the Tarsi Movement and the Tendons in the Ladybird Beetle, *Coccinella septempunctata*

### 3.1. The Leg Morphology of the Ladybird Beetle C. septempunctata

**Figure 2:**
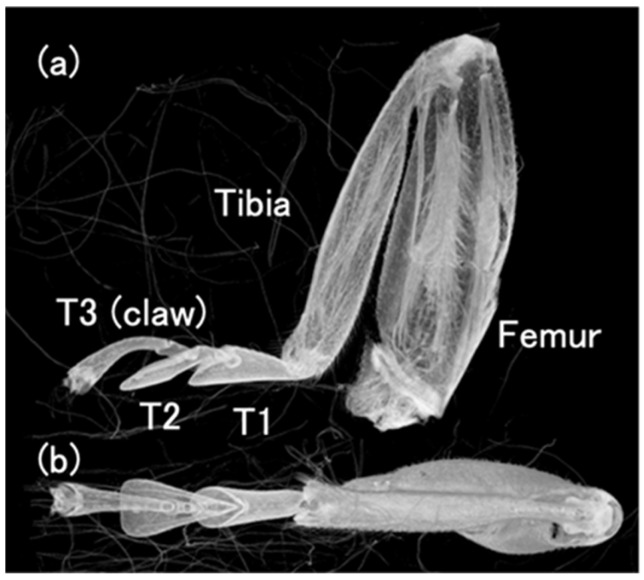
Left foreleg of ladybird beetle observed by micro-X-ray CT. (**a**) Side view and (**b**) top view. T3; Tarsal claw, T1 and T2; Tarsus.

**Figure 3:**
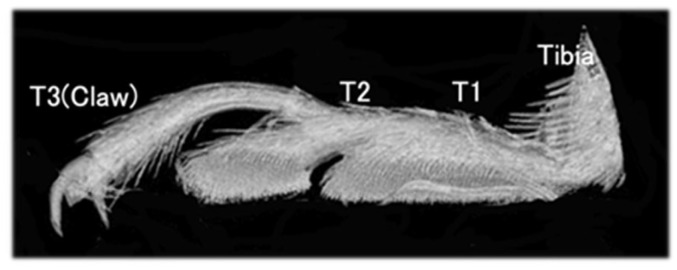
Overview of the tarsi connected to the base of the tibia.

At the end of the insect’s leg, there is an organ called the tarsus, which contacts on the ground. In the ladybird beetle, it consists of three segments, called the 1st tarsomere (T1), 2nd tarsomere (T2), and 3rd tarsomere with a claw (T3) at the end, from the tibial side, respectively. T1 and T2 have adhesive seta on the bottom. Figure 2 shows the left foreleg of a ladybird beetle observed by a 3D X-ray microscope. (a) Side view and (b) top view. T3; Tarsal claw, T1 and T2; Tarsus. Figure 3 shows an overview of the tarsi connected to the base of the tibia taken by a 3D X-ray microscope.

### 3.2. Movement of the Tarsal Segments of the Ladybird Beetle, Coccinella septempunctata, during Walking

**Figure 4:**
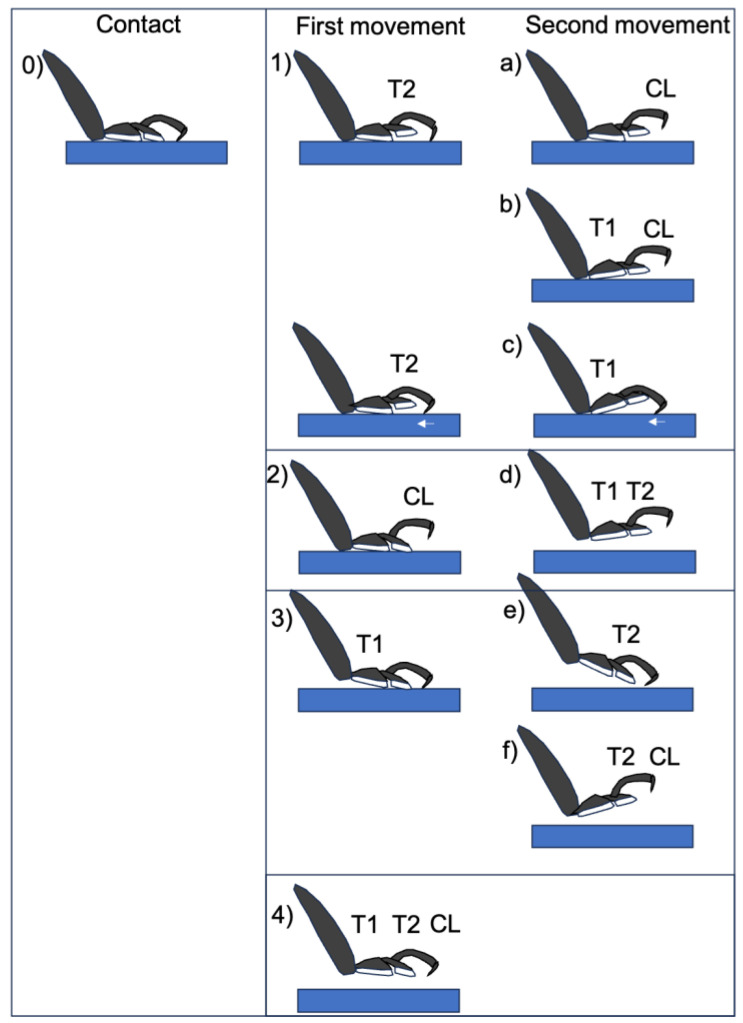
Type of movement of tarsi when ladybird legs are separated from the substrate. Figures show (**0**) state of contact, (**1**–**4**) first movement, (**a**–**f**) second movement. The symbols in the diagram are the locations where the detaching has occurred.

In order to develop a walking robot that imitates insects, the characteristics of the walking behavior of *Coccinella septempunctata* were investigated. To understand how adult ladybird beetles use their three-segmented tarsi to walk, a high-speed microscope was used to film the sequence of movements from the grounding of each tarsal on a smooth flat surface to its detachment. It could be classified into seven sequential patterns (Figure 4).

Many beetles do not place the entire surface of the T2 or T1 pads on the substrate, but rather use only 1/2 to 1/4 of the surface area to make a smooth centroid shift during walking.

**Figure 5:**
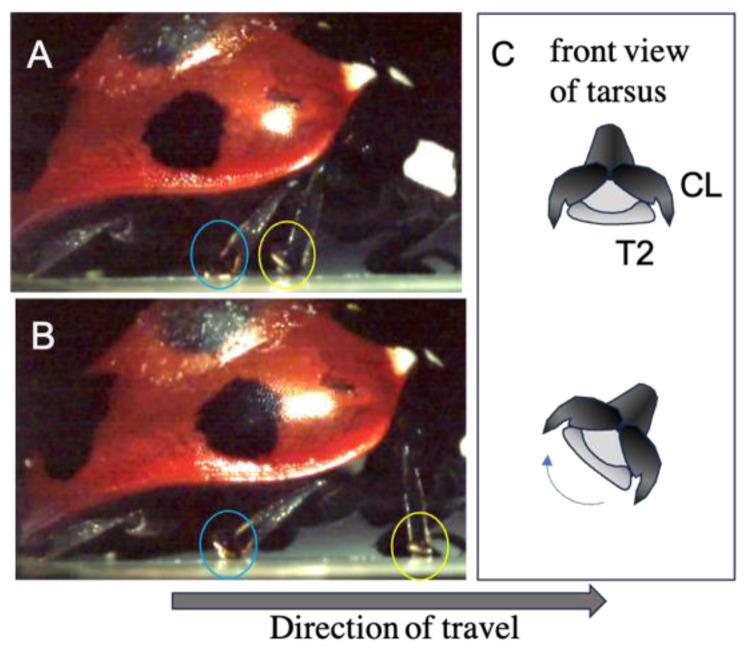
Movement of the tarsi when a ladybird walks from (**A**,**B**). The area circled in yellow is the right foreleg. The area circled in blue is the right middle leg. The ladybird walks by twisting the tarsi so that the side opposite to the direction of travel peels off first. The large tilt of the tarsus is linked to the tilt of the tibia associated with its movement. (**C**) is a schematic diagram of the tarsus movement of the middle leg circled in blue. The arrow shows the tarsi twisting movement.

**Figure 6:**
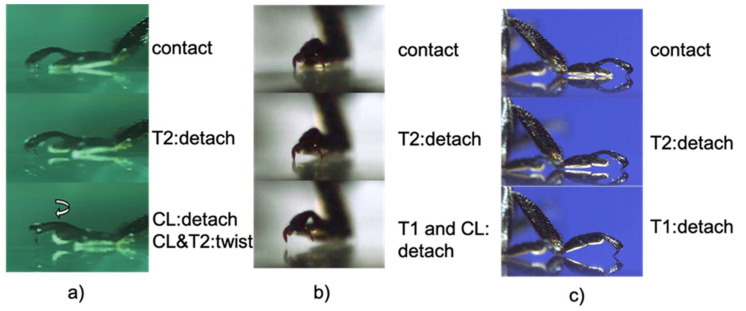
Photograph of detaching movement of (**a**): type (**a**), (**b**): type (**b**) and (**c**): type (**c**) in Figure 4a is an example of twisting T2 and claw. The arrow shows the tarsi twisting movement.

The initial movement of the legs was divided into four distinct types: (i) raising T2, (ii) raising T3, (iii) raising T1, and (iv) raising T1, T2, and T3 simultaneously. The second movement afterwards was of six types, as shown in Figure 4. In addition to these movements, twisting movements were also observed. The ladybird beetle walks by twisting the tarsi so that the side opposite to the direction of travel peels off first. The large tilt of the tarsus is linked to the tilt of the tibia associated with its movement (Figure 5). A photograph of the actual leg movement is shown in Figure 6.

### 3.3. Location of Tendons in Tarsi of Ladybird Beetle

The tendons in the tarsi were investigated from the base of the tibia to the claw. Four tendons were observed. Tendon 1 is located in the tibia, tendon 2 is connected to the claw from the tibia through T1, and tendon 3 is a double tendon and is connected to T2 from the tibia through T1.

**Figure 7:**
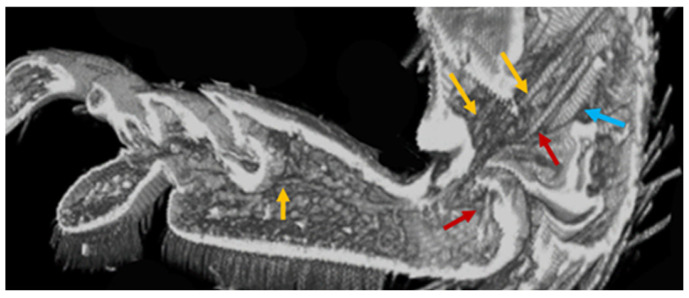
Tendon 1 (blue arrows), 2 (red arrows), and 3 (yellow arrows) at the joint between Tibia and T1.

**Figure 8:**
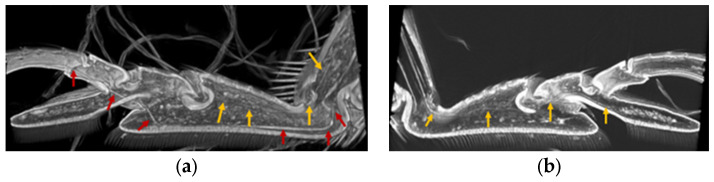
(**a**) Tendon 2 (red arrows) extending from Tibia to T3 through T1 and T2, as well as Tendon 3 (yellow arrows) running from T1 to T2. (**b**) is paired with (**a**) photo (cut and open left and right). (**b**) One of a pair of Tendon 3 from Tibia to T2 via T1.

**Figure 9:**
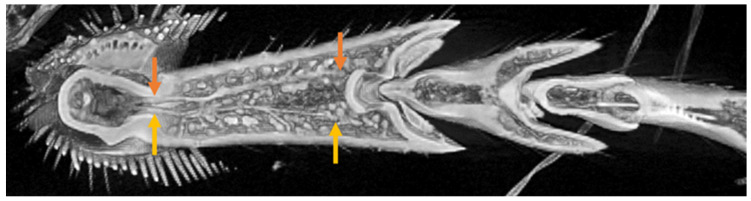
Double Tendon 3 (yellow and orange arrows) running through T1 from Tibia (bottom view).

**Figure 10:**
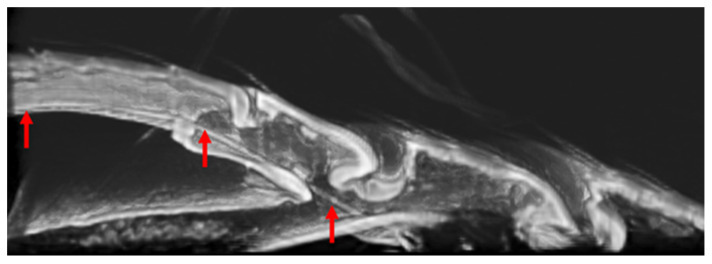
Tendon 2 (red arrows) running through T3 from T1 via the T2/T3 junction.

**Figure 11:**
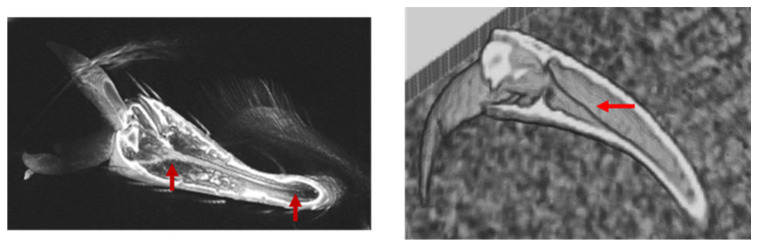
Tendon 2 (red arrows) extending to the base of the claw.

**Figure 12:**
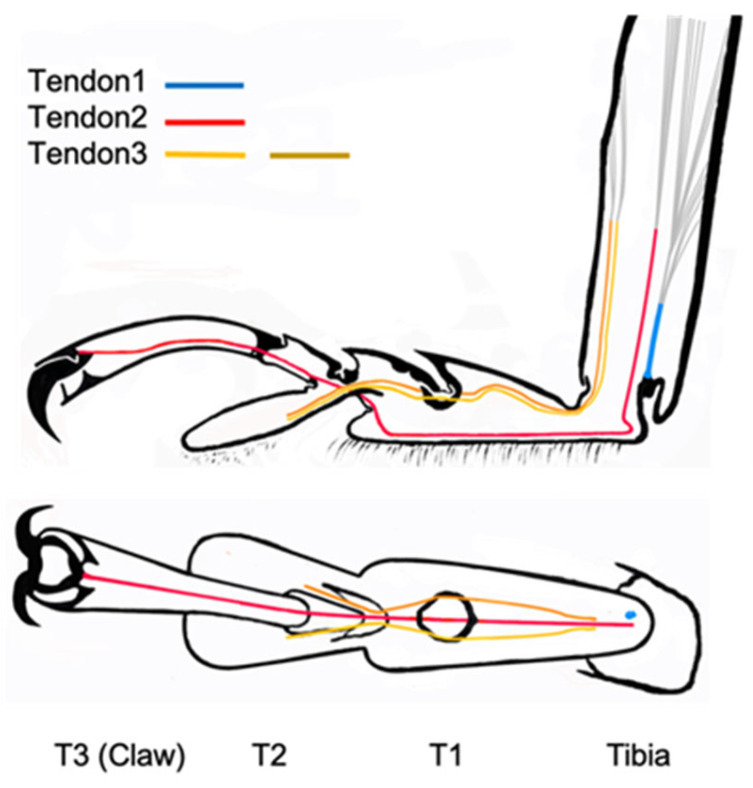
Schematic illustration of tendon positions inside the leg (side view and top view).

Figure 7, Figure 8, Figure 9, Figure 10 and Figure 11 show the photographs used to establish the location of the tendon in each tarsal segment. They are summarized as a schematic illustration in Figure 12.

### 3.4. Summary of the Role of Tendons on Tarsi Movements

Tendon 1 appears to control the opening and closing of the tibia/T1 joint by contracting and relaxing the muscles connected to it in the tibia. The vertical movement of T1 is controlled by tendon 1 (movement of T1 in Figure 4(d–f,3,4)). Tendon 2 may be involved in the vertical movement of T3 (and T2) including the claws. Therefore, T3 (and T2) is controlled by tendon 2 (movements T3 (and T2) in Figure 4(a–c,f,2,4).

The double Tendon 3 controls the movement of T2 and also the two claws by either pulling both of them at the same time, or only one of them to twist the tarsi. Therefore, the movements of T2 (Figure 4(b,f,1,4)) may be controlled by Tendon 3.

## 4. Design and Fabrication of Simplified Leg Structure

**Figure 13:**
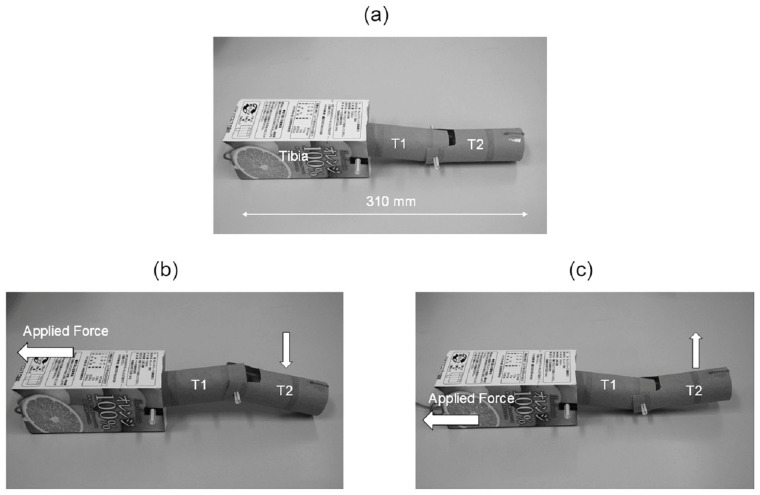
Basic cardboard pin-jointed model with (**a**) tarsus elements (T1 and T2); (**b**) T2 element moves downward followed by T1 (attachment); and (**c**) T2 moves upwards followed by T1 (detachment).

Following the study of the ladybird leg morphology and tendon arrangement detailed above, the next stage of the project was to design and fabricate a simplified structure based on the actual ladybird leg. Initially, a very basic model of the leg structure was constructed from cardboard tubes and drinking straws (Figure 13a). It was decided to replace the complex joints of the actual insect leg with simple pin joints for articulation as these were deemed sufficient. Thread was used to replicate the tendons in the insect leg. Analysis of the movement of the pin-jointed elements revealed two independent movement configurations for attachment and detachment of the tarsus elements from a substrate surface. These are illustrated in Figure 13b,c and approximately correspond to the movement configurations of the actual ladybird. The first configuration involves the initial downward movement of the tarsus element T2 (via rotation at the pin joint between T1 and T2) followed by T1 (via rotation at the pin joint between T1 and the tibia element). This will allow for attachment of the tarsus to surfaces and would be particularly useful for attachment to curved or cylindrical surfaces. The second movement configuration is the opposite and features the initial lifting of T2 followed by T1 to enable detachment of the tarsus from the substrate.

**Figure 14:**
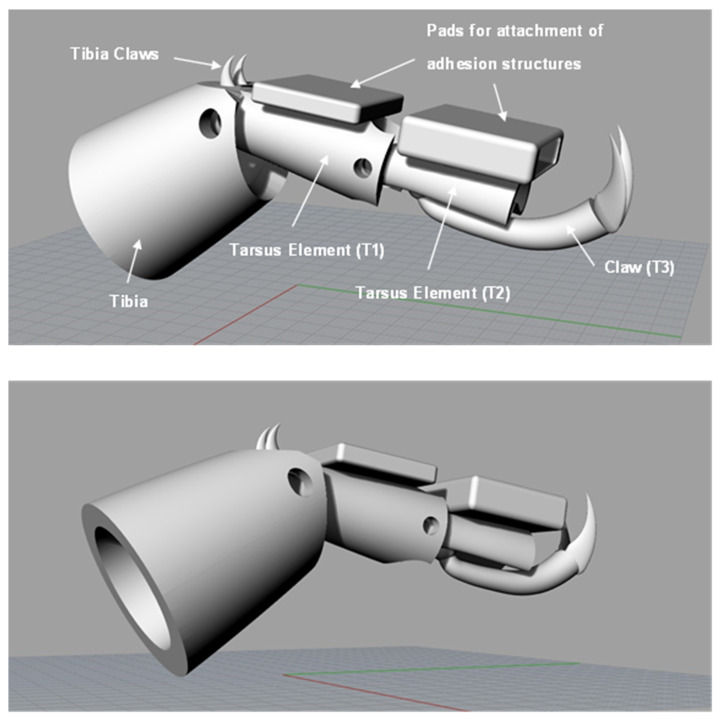
Final version of simplified ladybird leg structure design shown in CAD software.

Employing the knowledge gained from the cardboard model, a pin-jointed leg structure based on the studied ladybird leg was designed using the CAD software Rhinoceros 5 (Robert McNeel & Associates, Seattle, WA, USA). The design underwent a few iterations and modifications before the final version was realized and this version is shown in Figure 14. Square pads were added to the underside of the two tarsus elements for attachment of the PDMS adhesion structures.

Following the finalization of the simplified design, the individual parts of the simplified leg structure were fabricated from clear resin via 3-D printing and subsequently assembled. It was decided to fix the T3 claw element to the T2 tarsus element, as this was deemed sufficient for the intended attachment/detachment operation. The claws themselves (including the tibia claws) were fabricated from stainless steel via 3-D laser printing and affixed to the resin leg structure using cyanoacrylate adhesive. The PDMS adhesion structures were attached to the square pads on the T1 and T2 tarsus elements also using cyanoacrylate adhesive.

**Figure 15:**
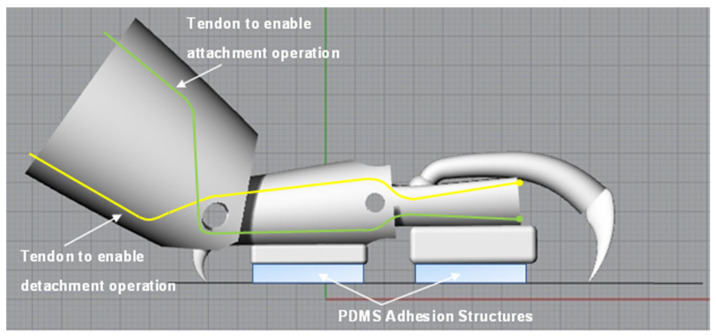
Schematic illustration showing configuration of tendons in final leg structure design.

**Figure 16:**
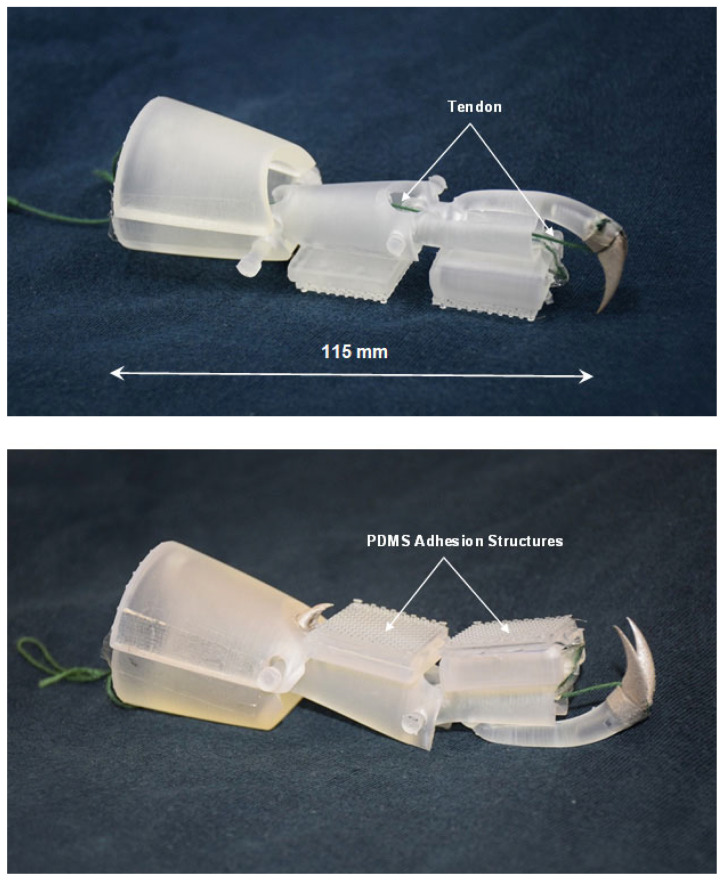
Final fabricated and assembled simplified ladybird leg structure.

Lastly, cord was used to replicate the tendons of the insect leg and was attached to the T2 tarsus element and threaded through guide rings placed at appropriate positions on the leg structure. A schematic illustration showing the configuration of the tendons in the simplified structure is shown in Figure 15. In this figure, the tendons are shown in yellow and green and approximately equate to Tendons 2 and 3 shown in Figure 12 for the actual insect leg. However, some modification of the tendon configuration was necessary in the case of the simplified leg structure to facilitate rotation at the pin-joints and to allow for the desired movement. Automated application of force to the tendons during operation to enable leg movement can be carried out using a remote-controlled motor or linear actuator. The final completed leg structure is shown in Figure 16.

**Figure 17:**
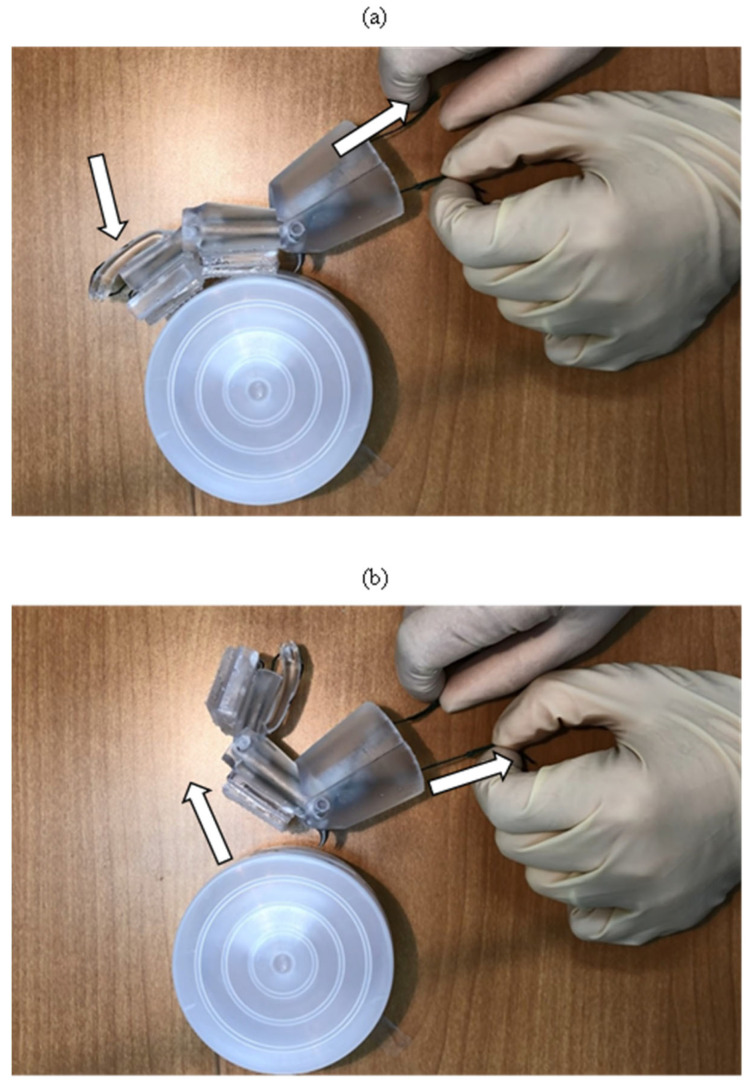
(**a**) Attachment of the tarsus elements to cylindrical object, (**b**) detachment occurring via manual manipulation of the tendons.

The movement configurations involved with the attachment and detachment of the T2 tarsus element to a vertical cylindrical object (via hand manipulation of the tendons) are presented in Figure 17 (and also in Supplementary Video S1: Attachment/detachment of Simplified Ladybird Leg Structure).

## 5. Experimental Procedures for Analysis of Ladybird Leg

**Figure 18:**
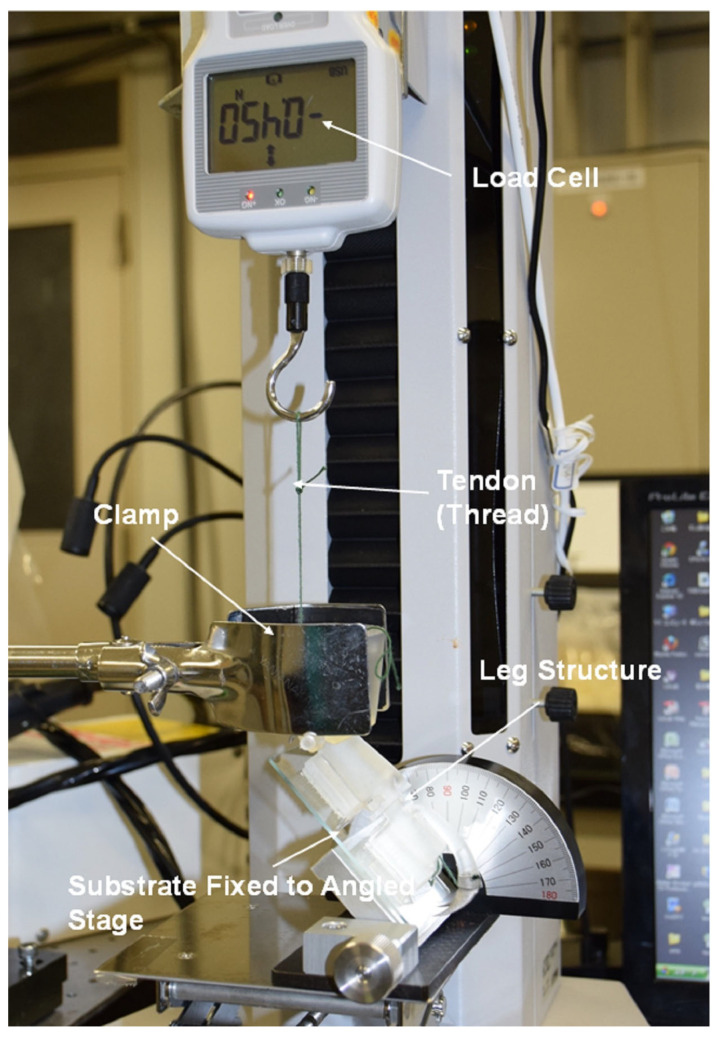
Experimental setup for measurement of adhesion/lifting forces.

Following fabrication and assembly of the simplified leg design and attachment of the adhesive structures to the two pads on the tarsus elements, experiments were conducted to measure the detachment forces from selected substrates using a small servo-electric tensile test machine (IMADA, Toyohashi, Japan). The arrangement of the leg structure during these experiments is shown in Figure 18. The tibia is held vertically in a clamp attached to a retort stand. The tarsus rests on an angled stage (typically at 45°) with the adhesive structures firmly attached to the test substrate. The substrate is fixed to the angled stage using double-sided tape. The crosshead was then moved upwards under displacement control (i.e. uni-axial tension), at a displacement rate of 25 mm/min, until detachment of the tarsus from the substrate occurred. The force was measured by a 50 N load cell (IMADA, Toyohashi, Japan) and recorded using ZP Recorder dedicated software. Initially, experiments were conducted with no adhesion to assess the force required to overcome gravity and friction and lift the tarsus elements (via rotation at the pin joints). Detachment force measurements were then performed using a glass microscope slide as a substrate which had been previously cleaned in an ultrasonic bath using acetone, ethanol, and then deionized water, sequentially. The effects of varying the angle of the tibia relative to the clamped substrate (by changing the angle of the stage) were also studied.

The potential applications of these leg structures may require the tarsus to attach to wet surfaces some of the time. Therefore, the detachment experiments were also conducted on a clean glass slide but this time with the presence of water droplets. The effect of the wet slide on the detachment forces was evaluated and compared to the performance measured on a dry substrate.

Finally, experiments were repeated using a soft (hardness: 50 Shore A) silicone rubber substrate (1 mm in thickness) attached to a glass slide and cleaned using the same procedure employed for the glass substrate. The primary reason for employing a soft substrate was to assess the ability of the claws (particularly those on the tibia element) to push into the surface and provide leverage or an anchoring function during the detachment operation.

## 6. Results and Discussion of Adhesion Force Experiments

**Figure 19:**
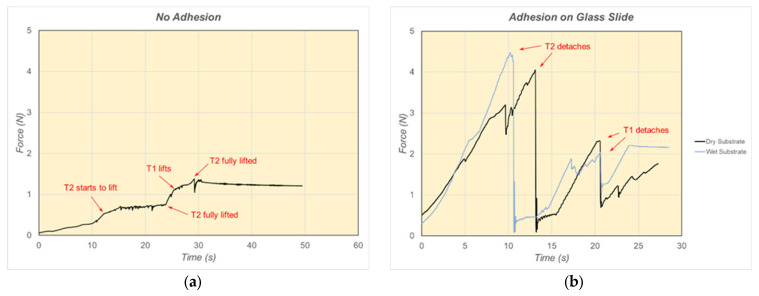
(**a**) Forces during lifting of tarsus elements; (**b**) detachment forces from (dry and wet) glass substrate.

Force-time plots for the structure with no adhesion and attached to a clean and dry glass slide (at an angle of 45°) are shown in Figure 19. As can be seen from Figure 19a, little force is required to overcome gravity and friction and lift the tarsus elements. The elements generally start to lift at a force of ~0.5 N or less. However, strong attachment to the glass substrate was achieved with adhesion structures applied to both tarsus elements. Typical detachment forces of approximately 4 N were measured (Figure 19b). This is an encouraging result since this level of adhesion could easily support the weight of a small robotic device. Varying the angle of the tarsus relative to the tibia between 20° and 70° was found to have little effect on the detachment force.

The effect of the presence of water droplets on the substrate is also shown in Figure 19b. It can be seen that the presence of water did not have a detrimental effect on the detachment forces. In fact, the forces measured for the wet substrate were generally higher than for the dry case. This is a desirable result since it implies that the function of the leg structure in outdoor applications will not be compromised by weather, moisture, etc.

**Figure 20:**
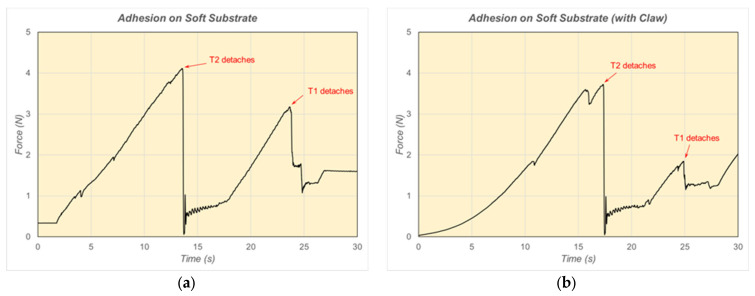
Detachment forces of tarsus elements from a soft rubber substrate. (**a**) no tibia claw; (**b**) tibia claw in contact with substrate.

Detachment forces up to about 4 N were also measured when using the soft silicone rubber substrate (Figure 20). The effect of the tibia claw can also be discerned from the plots in Figure 20. When the claw was not in contact with the soft substrate, the force exhibited a linear increase until detachment occurred (Figure 20a). On the other hand, when the tibia claw was in contact with the soft substrate, the force exhibited more of an exponential increase initially and resulted in somewhat longer times until detachment occurred (Figure 20b). It is surmised that the distribution of force between tibia claws and tarsus elements could account for this and may lead to a more controlled and stable detachment operation. Specifically, unless tibia claws are employed as an anchoring mechanism, the adhesion structure may pull (and elastically deform) a soft substrate in the lateral direction (elastic shear) when detachment forces are applied.

The average, maximum, and standard deviation of the detachment force measurements of the T2 tarsus element, based on at least five experiments for each substrate (dry glass, wet glass, and silicone rubber), are shown in Table 1.

## 7. Concluding Remarks

The 3D X-ray microscope and high-speed microscope analyses performed in this investigation have enabled a detailed understanding of the morphology and function of a ladybird leg. In particular, the location and configuration of the tendons that enable the movement of the leg elements have been closely studied. In this study, we showed for the first time that double tendons not found in flies and ladybirds are present in ladybirds and may contribute to the vertical movement or rotation of tarsomere T2 (and T3). The work has enabled the design and fabrication of a simplified version of the structure that utilizes pin-joints but nevertheless exhibits similar functionality and tendon configuration to the actual insect leg. With the subsequent attachment of special PDMS adhesion structures to the tarsus elements, strong adhesion forces of the tarsus pads to wet and dry substrates, (in some cases, over 6 N) were achieved. These values are more than sufficient to support the weight of a small robotic device. The leg structure movement then allowed for successful detachment from the substrate. Automated leg movement has been achieved using remote-controlled linear actuation. Based on these findings, proof of concept has been accomplished for the application of a simplified leg structure based on *Coccinella septempunctata* for attachment/detachment operations in small robotic devices.

## Figures and Tables

**Table 1:** Force Measurement Data for Detachment of T2 Tarsus Element.

Substrate	Maximum Force (N)	Average Force (N)	Standard Deviation
Dry Glass	4.4	3.6	0.8
Wet Glass	6.8	4.7	1.6
Soft Silicone Rubber	4.1	2.2	1.1

## Data Availability

The datasets presented in this article are not readily available because they are proprietary.

## Supplementary Materials

The following supporting information can be downloaded at https://www.mdpi.com/article/10.3390/biomimetics9030184/s1, Video S1: Attachment/detachment of Simplified Ladybird Leg Structure.
